# Generation of a functional fluorescently-tagged PFN-3 profilin transgene in *Caenorhabditis elegans*

**DOI:** 10.17912/micropub.biology.002134

**Published:** 2026-05-19

**Authors:** Michael Kimmich, David Pruyne

**Affiliations:** 1 Cell and Developmental Biology, SUNY Upstate Medical University, Syracuse, NY, US

## Abstract

Profilin is a small actin-monomer binding protein that inhibits pointed-end elongation and spontaneous nucleation, but promotes formin-mediated assembly of actin onto barbed-ends. In *Caenorhabditis elegans* , the profilin PFN-3 , in conjunction with the formin FHOD-1 , promotes sarcomere formation and dense body stability in the worm striated body-wall muscle (BWM). As a tool for further study of PFN-3 , we have expressed mScarlet-tagged PFN-3 (mSc::PFN-3) under *pfn-3* promoter sequence at a single exogenous locus. We demonstrate here that mSc::PFN-3 is expressed in multiple muscles, partially colocalizes with FHOD-1 in BWM, and rescues BWM defects associated with deletion of *pfn-3* .

**Figure 1. A functional fluorescently-tagged PFN-3 supports normal body-wall muscle cell growth and dense body morphogenesis:** **A** . Recombination template for MosSCI, including *mScarlet* and *pfn-3-* coding exons ( *red* for *mScarlet* , *black* for *pfn-3* ) and introns. **B** . Anti-mCherry western blot against extracts of independently isolated strains expressing free mScarlet or mSc::PFN-3. **C.** Montage of single confocal sections of a live L4 worm expressing mSc::PFN-3, showing expression in body-wall muscles (BWM), pharyngeal muscles (PM), vulval muscles (VM, inset), and the anal depressor (AD). **D.** Single confocal slice of a live L4 worm expressing mSc::PFN-3 in pharyngeal muscles PM3-PM8. **E.** Deconvolved confocal slices of a live L4 worm showing mSc::PFN-3 in BWM in the layer containing sarcomeres, and in the cytosolic layer overlying the sarcomeres. **F.** Deconvolved confocal slice showing a region of BWM of a fixed day-1 adult expressing mScar::PFN-3 and stained with fluorescently labeled phalloidin to show F-actin. Arrows indicate the locations of dot-like mSc::PFN-3 structures that coincide with gaps in F-actin indicative of dense bodies. **G.** Deconvolved confocal slice showing BWM of a live L4 worm expressing mSc::PFN-3 and FHOD-1::GFP. **H.** Maximum intensity projections of confocal slices show views of BWM of fixed day-1 adults of the indicated genotypes immunostained for dense body marker, ATN-1 . **I.** Quantification of percent area occupied by ATN-1 immunostain in views similar to **H** (n = 1 image per each of 10 animals per genotype, per each of three independent trials). **J.** Maximal widths of individual BWM cells from phalloidin-stained day-1 adults (n = two to four cells per each of 20 animals per genotype, per each of three independent trials). (ns) not significant; (**) *p* > 0.01; (***) *p* > 0.001; (****) *p* > 0.0001.

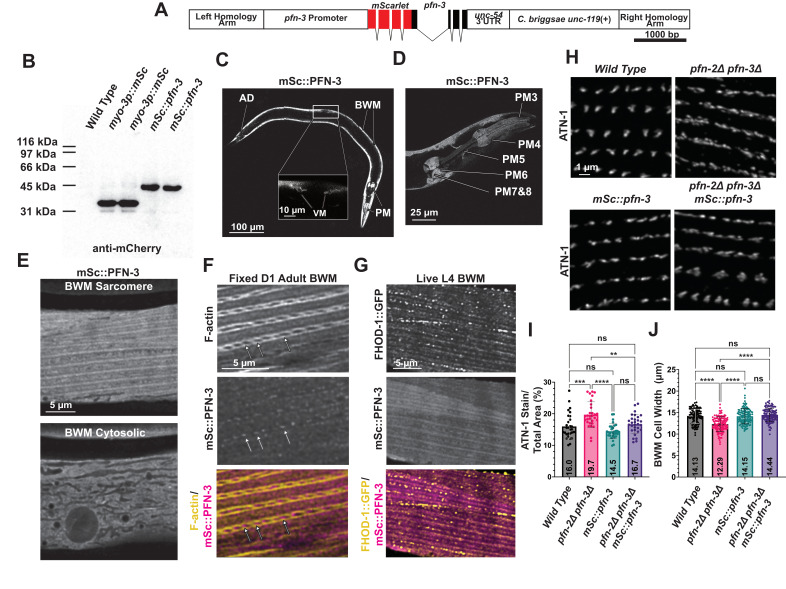



## Description

Profilin is a conserved protein that binds actin monomers in a 1:1 ratio, inhibiting spontaneous actin filament nucleation and monomer addition onto filament pointed-ends, but stimulating ADP/ATP exchange on actin, permitting monomer addition to filament barbed-ends, and enhancing formin-mediated actin polymerization (Kovar et al., 2003; Lu & Pollard, 2001; Moseley et al., 2004; Pollard et al., 2000; Pollard & Cooper, 1984; Pring et al., 1992; Romero et al., 2004). The nematode *Caenorhabditis elegans* encodes three profilin genes, *pfn-1* , *pfn-2* , and *pfn-3* . Neither *pfn-2* nor *pfn-3* is essential, but both impact muscle development, as their simultaneous deletion ( *pfn-2∆ pfn-3∆* ) results in modestly fewer but abnormally wide sarcomeres in striated body-wall muscle (BWM) (Kimmich et al., 2024; Polet et al., 2006), and their absence strongly exacerbates BWM filamentous actin (F-actin) defects in tropomodulin ( *unc-94* ) mutants (Yamashiro et al., 2008). Additionally, worms lacking *pfn-3* have misshapen and unstable dense bodies, structures that normally aid in anchoring thin filaments at sarcomere Z-lines in BWM (Kimmich et al., 2024). PFN-2 and PFN-3 proteins likely cooperate with the formin FHOD-1 , as worms lacking FHOD-1 , or expressing FHOD-1 missing its profilin-binding Formin Homology-1 domain, also have unstable dense bodies and fewer BWM sarcomeres (Kimmich et al., 2024; Mi-Mi et al., 2012).

Based on immunostain, PFN-3 was reported to localize to dot-like structures at the tips of dense bodies in BWM (Polet et al., 2006). With this anti-PFN-3 being no longer available (personal communications, S. Ono, Ph.D., Emory University), we set out to create a functional fluorescently-tagged PFN-3 . Amino (N)-terminal tagging of human profilin-1 with mApple and (SGLRSRAQAS) linker was shown to preserve a range of biochemical and cellular functions (Pimm et al., 2022). Thus, we set out to N-terminally tag PFN-3 with mScarlet and an identical linker. Our attempts to tag the endogenous *pfn-3* locus using CRISPR-Cas9 failed, but we successfully introduced a single copy *mScarlet::pfn-3* fusion transgene ( *mSc::pfn-3)* to a neutral locus on Chromosome II using the Mos-I transposon-mediated single copy insertion (MosSCI) technique (Frøkjær-Jensen et al., 2008). We included in this transgene 2023 bp *pfn-3* upstream sequence plus the first two codons of *pfn-3* upstream of *mScarlet::linker* , and the entire *pfn-3* coding region downstream of the linker, including all *pfn-3* introns and exons ( **Fig. 1 A** ). Two independent transgenic lines were isolated and validated by PCR (see Methods).

Western blot analysis of whole worm extracts using anti-mCherry verified expression of 41.2 kDa N-terminally-tagged PFN-3 (mSc::PFN-3), compared to 26.4 kDa free mScarlet expressed in control strains ( **Fig. 1 B** ). This 14.8 kDa difference is close to 13.5 kDa predicted for PFN-3 . In live worms of both independent mSc::PFN-3 lines, the fusion protein was visible in BWM, the anal depressor muscle (AD), and vulval muscles (VM) ( **Fig. 1 C** ), consistent with previous expression studies (Durham et al., 2021; Roux et al., 2023), as well as in pharyngeal muscles (PM), PM3 through PM8 ( **Fig. 1 D** ). Among the sarcomeres of BWM in live worms, mSc::PFN-3 was arranged in striations as well as irregular patches between striations ( **Fig. 1 E, BWM Sarcomere** ), while in the cytosol overlying the sarcomeres, mSc::PFN-3 appeared diffuse ( **Fig. 1 E, BWM Cytosolic** ). We did not observe dot-like mSc::PFN-3 structures in live animals, but in fixed worms stained with fluorescent phalloidin to show F-actin, we sometimes observed mSc::PFN-3-positive dots associated with gaps in F-actin indicative of dense bodies ( **Fig. 1 F, *arrows*** ). We suggest dense body-associated mSc::PFN-3 is likely present as was indicated by immunostain (Polet et al., 2006), but this is obscured in live animals by additional diffuse or loosely bound mSc::PFN-3 that is lost during fixation and staining.

To compare PFN-3 and FHOD-1 localizations, we crossed *mSc::pfn-3* into a background expressing functional FHOD-1 bearing a carboxyl-terminal GFP tag (FHOD-1::GFP) (Mi-Mi et al., 2012). FHOD-1::GFP normally occupies bright puncta and patchy striations in BWM (Mi-Mi et al., 2012; Sundaramurthy et al., 2020). In live animals, FHOD-1::GFP extensively overlapped with mSc::PFN-3, but, to within our limits of detection, there were also some areas of non-overlap ( **Fig. 1 G** ). This supports the possibility that PFN-3 and FHOD-1 interact within the sarcomeres to promote sarcomere growth and proper dense body morphogenesis, while areas of non-overlap may mark an inactive pool of FHOD-1 and/or FHOD-1 acting independently of PFN-3 .

To test whether mSc::PFN-3 is functional, we crossed *mSc::pfn-3* into a *pfn-2∆ pfn-3∆* background. In wild-type worms, dense bodies are semi-regular in shape and spacing along striations, while dense bodies in *pfn-2∆ pfn-3∆* mutants appear irregular (Kimmich et al., 2024). This defect can be quantified by measuring the percent area occupied by the dense body marker a-actinin/ ATN-1 in a field of view, as dense body malformation increases the relative area occupied by ATN-1 (Kimmich et al., 2024). Based on analysis of ATN-1 immunostain, mSc::PFN-3 expression restored normal dense body morphology to *pfn-2∆ pfn-3* ∆ worms ( **Fig. 1 H & I** ). *pfn-2∆ pfn-3∆* mutants have a very modest but statistically significant reduction in the width of BWM cells, which correlates with the presence of fewer sarcomeres per cell (Kimmich et al., 2024; Mi-Mi et al., 2012). When we measured the width of phalloidin-stained BWM cells, again we observed mSc::PFN-3 rescued *pfn-2∆ pfn-3* ∆ mutant defects ( **Fig. 1 J** ). Additionally, we observed no dense body or cell size defects in otherwise wild-type animals expressing mSc::PFN-3 ( **Fig. 1 H-J** ), suggesting the *mSc::pfn-3* transgene itself does not cause muscle defects.

Our results show mSc::PFN-3 is functional with regard to sarcomere development in BWM, making it a useful tool for studying the muscle-specific functions of this profilin family member.

## Methods

Plasmids

Plasmid pKM7 with *mSc::pfn-3* was generated using In-Fusion cloning (Takara Bio USA, San Diego, CA) to combine: 2023 bp *pfn-3* upstream sequence (PCR-amplified from N2 genomic DNA); worm codon-optimized *mScarlet* with 10-amino acid SGLRSRAQAS linker (PCR-amplified from LP896 genomic DNA); *pfn-3* coding region including introns and exons (from N2 genomic DNA); and vector backbone (from pCFJ90). *lim-6* is positioned almost immediately downstream of *pfn-3* , with an opposite orientation. In the hopes of avoiding induction of RNA interference against *lim-6* , we chose to not include *pfn-3* downstream sequence. Instead, we utilized the *unc-54* 3'UTR already present in pCFJ90, as this 3'UTR will support expression in most, if not all, somatic tissues (Mello and Fire, 1995). MosSCI recombination template plasmid pMK14 was generated by In-Fusion cloning *mSc::pfn-3* (from pKM7) into pCFJ151. Plasmid pKM16 expressing free mScarlet was generated using In-Fusion cloning to replace *mCherry* in BamHI/EcoRI-linearized pCFJ104 with *mScarlet* . All plasmid sequences were verified by next generation whole-plasmid sequencing (Genewiz, South Plainfield, NJ).

Worm Strains and Growth Conditions

Worms were maintained on nematode growth medium agar with OP50-1 bacteria food at 20°C (Brenner, 1974). MosSCI (Frøkjær-Jensen et al., 2008) was performed by gonadal microinjection of EG4322 adults with pKM14 (50 ng/µl), transposase-expression vector pJL43.1 (50 ng/µl), and GFP-expressing pDP401 (5 ng/µl), pSDV30 (2.5 ng/µl) and pSDV31 (5 ng/µl). Progeny of injected animals were grown to starvation and then screened for normal motility (from *unc-119 (+)* in pKM14) and absence of GFP (which otherwise would indicate the presence of an extrachromosomal array). Insertion at the *ttTi5065* locus was confirmed by PCR-based detection of upstream and downstream insertion junctions, and failed detection of unmodified *ttTi5065* locus. *mSc::pfn-3* coding regions were sequenced from two independent isolates ( DWP359 , DWP360 ) to confirm absence of mutations. *mSc::pfn-3* was outcrossed from DWP359 into a clean N2 background before crossing into DWP3 with *fhod-1::gfp* (resultant DWP396 ), or into DWP374 with *pfn-2 (∆) pfn-3 (∆)* (resultant DWP391 ). For soluble mScarlet expression, N2 adults were gonadally microinjected with pKM16 (5 ng/µl), pSDV30 (2.5 ng/µl), pDP401 (10 ng/µl), and pRS315 (82.5 ng/µl), and two independent mScarlet- and GFP-positive transformants were isolated ( DWP371 , DWP372 ).

Western blot analysis

Worms were collected and lysed as described previously (Yingling & Pruyne, 2021). Sample loads were normalized based on whole-lane Coomassie brilliant blue stain of worm extracts after SDS-PAGE. Nitrocellulose blots of normalized extracts were probed with Living Colors mCherry Monoclonal Antibody (diluted 1:1000) and peroxidase-conjugated goat anti-mouse (diluted 1:3000), as described previously (Mi-Mi et al., 2012).

Microscopy

Worms were fixed and stained with Alexa Fluor 488-phalloidin (diluted 1:250) to detect F-actin, or with antibody MH35 (diluted 1:10,000) to detect ATN-1 and FITC-conjugated goat anti-mouse (diluted 1:500), as previously (Kimmich et al., 2024). Live worms were immobilized for microscopy in 3.5 µL of 0.1 µm-diameter polystyrene nanobead suspension (Polysciences, Warrington, PA) between a coverslip and 10% agarose pad, as previously (Kimmich et al., 2024). Fixed or live worms were imaged on an SP8 Laser Scanning Confocal Microscope (Leica, Wetzlar, Germany) using LAS X software (version 3.5.2, build 4758; Leica) and HCX plan apochromat 63x/NA 1.4 oil immersion lambda objective. Deconvolution was performed using Huygens Essential software (Huygens Compute Engine 18.10.0, Scientific Volume Imaging, Hilversum, Netherlands). Images were false colored and subject to linear brightness adjustment using Adobe Photoshop CS4 (Adobe, San Jose, CA). Widths of BWM cells in phalloidin-stained day-1 adults were measured for two to four cells in each of 20 animals, in each of three independent replicate experiments, as previously (Kimmich et al., 2024; Mi-Mi et al., 2012; Sundaramurthy et al., 2020). Quantification of ATN-1 immunostain area in day-1 adults was performed for one image with 4-6 striations for each of 10 animals, in each of three independent replicate experiments, as previously (Kimmich et al., 2024). Analyses were performed blinded to strain identity.

&nbsp;

Statistical Analysis

Graphs were designed in Prism 10 (version 10.1.1 **;** GraphPad Software, Boston, MA). Data were analyzed using one-way analysis of variation with a Tukey's multiple comparison *post hoc.p* < 0.05was considered statistically significant. Data are shown as mean ± SD, and as individual measurements.

## Reagents

**Table:** 

**Plasmid**	**Contents**	**Source**
pRS315	budding yeast/bacteria shuttle vector	Sikorski and Hieter, 1989&nbsp;
pCFJ90	*myo-2p::mCherry::unc-54 3' UTR*	Addgene Plasmid #19327
pCFJ104	*myo-3p:mCherry::unc-54 3' UTR*	Addgene Plasmid #19328
pCFJ151	*ttTi5605* -targeting region with *C. briggsae unc-119* casette	Addgene Plasmid #19330
pJL43.1	*glh-2p::Mos1 transposase::glh-2 3' UTR*	Addgene Plasmid #19332
pDP401	*rab-3p::gfp::unc-54 3' UTR*	Sundaramurthy et al., 2020
pSDV30	*myo-2p::gfp::unc-54 3'UTR*	Sundaramurthy et al., 2020
pSDV31	*myo-3p::gfp::unc-45 3' UTR*	Sundaramurthy et al., 2020
pKM7	*pfn-3p::mScarlet::pfn-3::unc-54 3' UTR*	This study
pKM14	*pfn-3p::mScarlet::pfn-3* + *C. briggsae unc-119* + *ttTi5605* homology arms	This study
pKM16	*myo-3p::mScarlet::unc-54 3' UTR*	This study
		
**Primer**	**Sequence (5' to 3')**	**Products**
**Genotyping Primers**		
MosSCI - ttTi5605 Fwd	GTT-GTC-GAC-CTA-CAA-AAT-TTG	2.1 kb product with successful MosSCI
MosSCI - Pfn-3 Promoter Rev	GCT-TTT-TAG-GAC-GGC-TCA-TTT-TG	
MosSci- unc-119 Fwd	CTA-ACT-TTG-AGC-CAA-TTC-ATC-CC	1.8 kb product with successful MosSCI
MosSci- ttTi5605 Rev	CTC-TGC-TTC-TTC-GTT-GTA-CCA-T	
MosSci RHA Fwd	GTA-CTA-ATA-AAT-GCA-AGA-CAC	1.2 kb product for undisturbed ttTi5065 locus
MosSci LHA Rev	CCG-AAA-TTT-TAC-TTG-ATC-GAG	
**Primers to add linker to mSc**		
mScarlet Repair Fwd	ACT-TTC-AAA-GTT-TTC-AGC-ACA-CAC-AAA-ACG-AAA-ACA-TGT-CGA-TGG-TCT-CCA-AGG-GAG-AGG-C	mScarlet + SGLRSRAQAS linker
mScarlet Repair Rev	ATA-AGG-TTA-TTG-TTG-ATA-ATA-TCA-GAC-CAC-GAC-ATT-GAT-GCT-TGT-GCA-CGT-GAA-CGC-AGG-CCG-CTC-TTG-TAG-AGC-TCG-TCC-ATT	
**In-Fusion Cloning Primers**		
Pfn3Promoter-INFU-Fwd	TGT-ATA-GAA-AAG-TTG-ATC-AAC-AAA-TCA-ACT-GTA-CCA-AC	pfn-3 promoter for pMK7
Pfn3Promoter-INFU-Rev	GGA-GAC-CAT-TCG-CAT-GTT-TTC-GTT-TTG-TGT-GTG-CTG	
Pfn3gDNA-INFU-Fwd	CGT-GCA-CAA-GCA-TCA-ATG-TCG-TGG-TCT-GAT-ATT-ATC	pfn-3 coding region for pMK7
Pfn3gDNA-INFU-Rev	ACA-AGA-AAG-CTG-GGT-TCA-GTA-CTT-GAT-GGA-CCT-GAA-GTA-A	
mScar-INFU-Fwd	ACA-CAA-AAC-GAA-AAC-ATG-CGA-ATG-GTC-TCC-AAG-GG	mScarlet + linker for pMK7
mScar-INFU-Rev	ATC-AGA-CCA-CGA-CAT-TGA-TGC-TTG-TGC-ACG-TGA-AC	
Vector -INF-Fwd	TCC-ATC-AAG-TAC-TGA-ACC-CAG-CTT-TCT-TGT-ACA-AAG-TG	pCFJ90 backbone for pMK7
Vector INF-New Rev	TTG-CGA-CGT-TTT-GCC-CAA-CTT-TTC-TAT-ACA-AAG-TTG	
mScar Pfn3 MosSCI New Fwd	GCG-CGC-ACC-GTA-CGT-CTC-GAA-TCA-ACA-AAT-CAA-CTG-TAC-CAA-CTC-TG	pfn-3p::mSc::pfn-3 for pMK14
MosSci mScar Pfn-3 New Rev	TCC-TGC-AGG-AAT-TCC-TCG-AGG-AAA-CAG-TTA-TGT-TTG-GTA-TAT-TGG-G	
pCFJ151 vector Fwd	CTG-CAG-GAT-ATC-TGG-ATC-CAC-G	pCFJ151 vector for pMK14
pCFJ151 vector Rev	GAA-TTC-CTC-GAG-ACG-TAC-GGT	
pMyo3-mScar-INF-F	GCA-GGC-TTA-GGA-TCC-ATG-GTC-TCC-AAG-GGA-GAG-GC	mScarlet with stop codon for pMK16
pMyo3-mScar-INF-R	AAG-CTG-GGT-GAA-TTC-CTA-CTT-GTA-GAG-CTC-GTC-CAT-TCC	
		
**Strain**	**Genotype**	**Source**
N2	Bristol Wild Type *Caenorhabditis elegans*	CGC
EG4322	*ttTi5605 II; unc-119 ( ed4 ) III *	A gift from E. Maine (Syracuse University, Syracuse, NY)
LP896	*unc-94 ( cp439 [unc-94::mNG-C1)]) I; cap-1 ( cp436 [mScarlet-I-C1::cap-1)]) IV *	A gift from B. Goldstein and Z. Pu (University of North Carolina Chapel Hill, Chapel Hill, NC)&nbsp;
DWP3	*qaIs8001 [fhod-1::gfp mini- unc-119 (+)] *	Mi-Mi et al., 2012
DWP292	*pfn-3 ( tm1362 ) pfn-2 ( ok458 ) X *	Kimmich et al., 2024
DWP359	*upsIs219 [pfn-3p::mScarlet::pfn-3::unc-54 3' UTR + C. b. unc-119 (+)] II; unc-119 ( ed4 ) III *	MosSCI pKM13 into EG4322
DWP360	*upsIs220 [pfn-3p::mScarlet::pfn-3::unc-54 3' UTR + C. b. unc-119 (+)] II; unc-119 ( ed4 ) III *	MosSCI pKM13 into EG4322
DWP371	*upsEx230 [myo-3p::mScarlet myo-2p::gfp rab3-p::gfp] *	Microinjection of pKM16 into N2
DWP372	*upsEx231 [myo-3p::mScarlet myo-2p::gfp rab-3p::gfp] *	Microinjection of pKM16 into N2
DWP374	*upsIs219 [pfn-3p::mScarlet::pfn-3::unc-54 3' UTR + C. b. unc-119 (+)] II *	DWP 359 outcrossed&nbsp; to N2 6 times
DWP391	*upsIs219 [pfn-3p::mScarlet::pfn-3::unc-54 3' UTR + C. b. unc-119 (+)] II; pfn-3 ( tm1362 ) pfn-2 ( ok458 ) X *	DWP374 X DWP292
DWP396	*upsIs219 [pfn-3p::mScarlet::pfn-3::unc-54 3' UTR + unc-119 (+)] II; qaIs8001 [fhod-1::gfp + mini- unc-119 (+)] *	DWP3 X DWP374
		
**Antibody**	**Details**	**Source**
MH35	target: ATN-1 ; mouse monoclonal	R.H. Waterston (Francis and Waterston, 1985), a gift from P. Hoppe (Western Michigan University, Kalamazoo, MI)&nbsp;
Fluorescein conjugated goat anti-mouse	target: mouse IgG (H&L); goat polyclonal, fluorescein-conjugated	Rockland, Cat #610-102-121
Living Colors mCherry Monoclonal Antibody	target: mCherry; mouse monoclonal	Clontech Laboratories, Cat. #632543
Peroxidase conjugated goat anti-mouse	target: mouse IgG + IgM (H&L); goat polyclonal; peroxidase-conjugated	Rockland, Cat #610-103-115
